# Immune-related molecular links between asthma and Alzheimer’s disease identified by multi-omics, machine learning and single-cell analysis

**DOI:** 10.3389/fcell.2026.1907664

**Published:** 2026-09-28

**Authors:** Li-jie Yang, Ni-shan Deng, Miao-juan Zhu, Xiu Shi, Jiong Yang, Han-Xiang Nie

**Affiliations:** 1 Department of Respiratory and Critical Care Medicine, Renmin Hospital of Wuhan University, Wuhan, China; 2 Department of Respiratory and Critical Care Medicine, Zhongnan Hospital of Wuhan University, Wuhan, China; 3 Hubei Key Laboratory of Metabolic and Chronic Diseases, Renmin Hospital of Wuhan University, Wuhan, China

**Keywords:** Alzheimer’s disease, asthma, immune regulation, machine learning, multi-omics, single-cell RNA sequencing

## Abstract

**Objective:**

Epidemiological evidence suggests that asthma increases the risk of Alzheimer’s disease (AD), yet their shared immune-related molecular mechanisms remain unclear. This study aimed to identify immune-associated molecular links between the two diseases.

**Methods:**

Gene expression datasets from GEO were analyzed to identify differentially expressed genes (DEGs) and enriched pathways. WGCNA was used to detect immune-related modules, and machine learning algorithms were applied to refine candidate biomarkers. Mendelian randomization (MR) and experimental validation were conducted to investigate genetic associations and expression correlations. A logistic regression model and nomogram were constructed. Immune characteristics were further explored using GSEA, immune-cell signature analysis, and single-cell RNA sequencing (scRNA-seq).

**Results:**

CD163 and CHST6 were identified as cross-disease candidate genes. MR analyses showed nominal genetic associations of CD163 expression and plasma protein levels with asthma- and AD-related phenotypes, although these associations did not remain significant after FDR correction. CD163 and CHST6 also showed increased expression in independent asthma and AD mouse models. The combined diagnostic model showed good discrimination (AUC >0.87). Immune-cell signature analysis revealed tissue-dependent associations between the candidate genes and immune-related signatures. scRNA-seq revealed cell-type-specific expression patterns, with CD163 predominantly localized to myeloid-lineage populations, including microglia in AD and monocytes in severe asthma, whereas CHST6 showed context-dependent localization across glial and airway epithelial populations.

**Conclusion:**

CD163 and CHST6 represent potential cross-disease molecular candidates associated with asthma and AD. CD163 may reflect a systemic immune-associated signal, whereas CHST6 may reflect tissue- and context-dependent cellular changes. These findings provide a preliminary framework for further investigation of asthma–AD comorbidity and require validation in prospective and functional studies.

## 1 Introduction

The pathology of Alzheimer’s disease (AD) extends beyond the classic amyloid-β and Tau hypotheses. In recent years, neuroinflammation has been widely recognized as a central driver of AD pathogenesis (Botella Lucena and Heneka, 2024). The central nervous system, traditionally considered relatively independent, has now been shown to maintain dynamic bidirectional interactions with the peripheral immune system (Ma et al., 2025). This “brain–body axis” framework suggests that chronic inflammation in peripheral tissues can exacerbate neuroinflammation, thereby promoting neurodegeneration and increasing the risk of AD (Vandal et al., 2025).

Clinical evidence reveals a high prevalence (44.1%) of cognitive impairment in adults with asthma, with longer disease duration associated with greater dysfunction (Rhyou and Nam, 2021). This association underscores that asthma is not merely a pulmonary disease but a systemic inflammatory disorder. Its characteristic type 2 (Th2) inflammation involves the systemic release of cytokines (e.g., IL-4, IL-5, IL-13), leading to a persistent low-grade inflammatory state (Howell et al., 2023). The “lung–brain axis” hypothesis provides a framework for understanding how this systemic inflammation may elevate AD risk (Joh et al., 2023). Mechanistically, this crosstalk is mediated through multiple pathways: systemic inflammatory mediators can cross a compromised blood–brain barrier and activate microglia (Rhyou and Nam, 2021); allergen exposure can stimulate brainstem noradrenergic neurons, forming neural connections (Su et al., 2024); and shared neuroendocrine signaling provides a biological link between stress and neuroinflammation (Dill-McFarland et al., 2024).

Despite accumulating clinical and mechanistic evidence, the specific molecular biomarkers that connect asthma and AD remain poorly defined. While our previous bioinformatic study focused on identifying asthma-specific diagnostic biomarkers (Yang et al., 2025), the present study extends this work to address their comorbidity. Here, we aim to identify shared immune-related molecular biomarkers between asthma and AD by integrating multi-omics analysis, machine learning, single-cell sequencing, and experimental validation, thereby providing insights into their potential immunological linkage and candidates for further investigation in asthmatic patients.

## 2 Materials and methods

### 2.1 Data acquisition

Six gene expression datasets were retrieved from the GEO database (https://www.ncbi.nlm.nih.gov/geo/). The selected asthma-related datasets included GSE143303 (endobronchial biopsies, *N*asthma = 47, *N*control = 13, training cohort), GSE147878 (endobronchial biopsies, *N*asthma = 59, *N*control = 13, validation cohort), and GSE69683 (peripheral blood, *N*asthma = 323, *N*control = 87, validation cohort). The selected AD-related datasets include GSE29378 (brain, *N*AD = 31, *N*control = 32, training cohort), GSE5281 (brain, *N*AD = 87, *N*control = 74, validation cohort), and GSE63061 (peripheral blood, *N*AD = 131, *N*control = 133, validation cohort). The detailed information of these datasets is shown in Table 1. The analysis workflow of this study is shown in Figure 1.

**TABLE 1:** Detailed information on datasets used in the study.

Diseases	Datasets	Platforms	Sample size		Organism	Tissue
			Control treat			
Asthma	GSE143303	GPL10558	13	47	*Homo sapiens*	Endobronchial biopsies
	GSE147878	GPL10558	13	59	*Homo sapiens*	Endobronchial biopsies
	GSE69683	GPL13158	87	323	*Homo sapiens*	Peripheral blood
AD	GSE29378	GPL6947	32	31	*Homo sapiens*	Brain
	GSE5281	GPL570	74	87	*Homo sapiens*	Brain
	GSE63061	GPL10558	133	131	*Homo sapiens*	Peripheral blood

**FIGURE 1:**
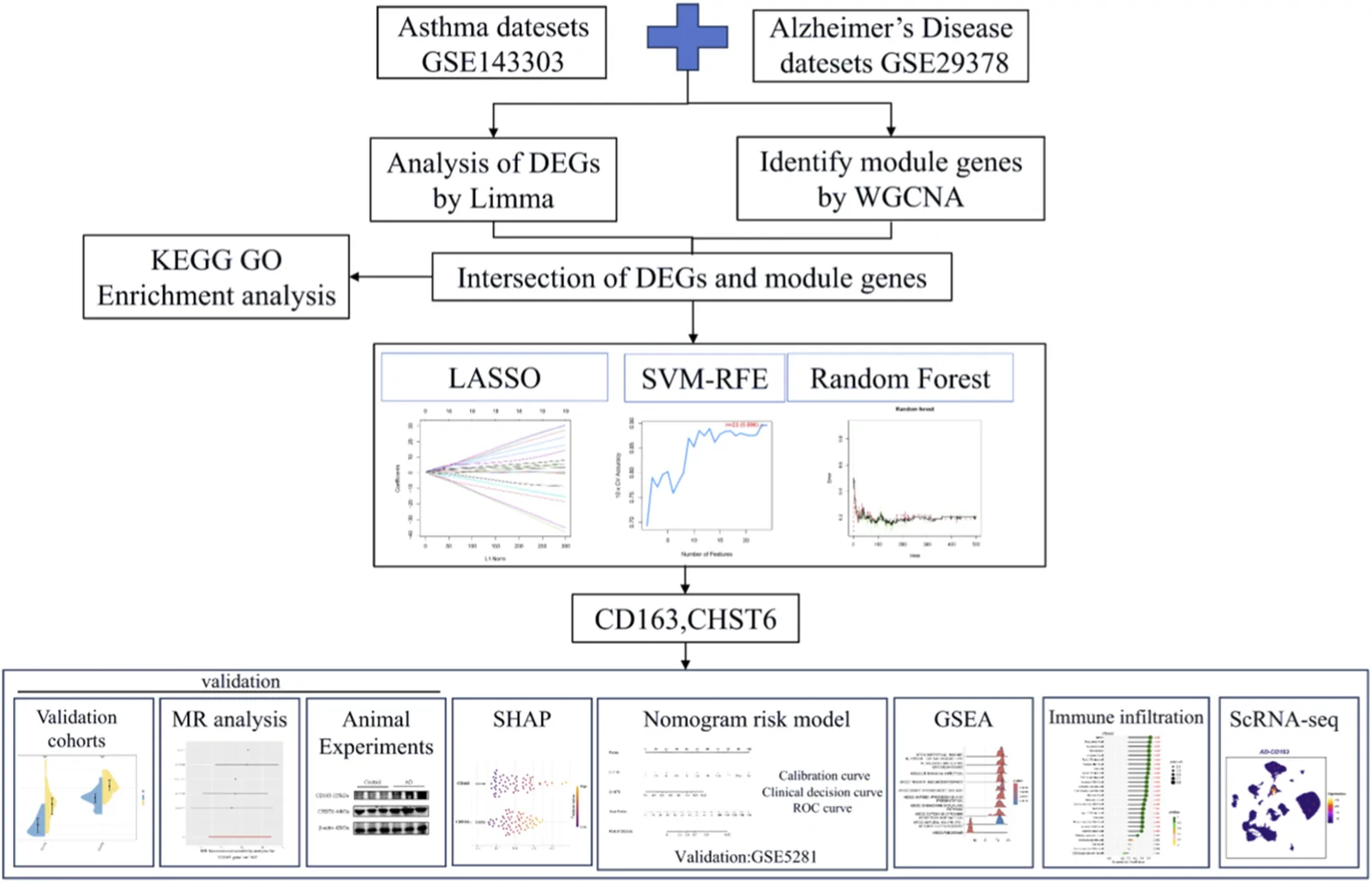
The flowchart of this study.

Each GEO dataset was preprocessed and analyzed independently, and no direct cross-platform merging of airway and brain expression matrices was performed. Shared candidate genes were identified only after dataset-specific differential-expression and network analyses.

### 2.2 Mendelian randomization analysis

A two-sample MR analysis (Yan et al., 2025) was used to investigate potential genetic associations between asthma and AD. Our MR design relied on three core assumptions: relevance, independence, and exclusion restriction. In the primary analysis, summary-level GWAS data for asthma (exposure) were obtained from the FinnGen consortium, while data for AD (outcome) were sourced from the IGAP consortium. To further evaluate the genetic associations of CD163 with asthma- and AD-related phenotypes, additional MR analyses were performed using CD163 plasma protein levels (pQTL data from the UK Biobank [ukb-ppp-CD163-OID20360]) and CD163 gene expression derived from the eQTLGen whole-blood eQTL dataset (eqtl-a-ENSG00000177575), with asthma and AD from FinnGen as outcomes. Detailed information regarding sample sizes, case/control ratios, and specific dataset IDs is summarized in Supplementary Tables S1–S4. Since all GWAS summary data are publicly available, ethical approval was obtained by original studies and further ethical review was waived.

For the selection of genetic variants, single nucleotide polymorphisms (SNPs) strongly associated with the exposures were extracted at a genome-wide significance threshold (*P* < 5 × 10^−8^). Linkage disequilibrium (LD) clumping was performed (r^2^ < 0.001, clumping window = 10,000 kb) to ensure instrument independence, and SNPs with an F-statistic <10 were excluded. All MR analyses were conducted with the “TwoSampleMR” R package, applying the inverse variance weighted (IVW) method as the primary approach.

Exposure and outcome datasets were harmonized according to effect alleles before MR analysis. Palindromic SNPs with ambiguous strand orientation were excluded, whereas allele-frequency information was used to infer strand orientation only when alignment was unambiguous. Instrument strength was assessed using the F-statistic, and the variance explained by the genetic instruments was estimated using R^2^. Cochran’s Q test was used to assess heterogeneity, and the MR-Egger intercept test was used to evaluate directional horizontal pleiotropy. Leave-one-out analyses were performed to assess the influence of individual variants. Detailed instrument characteristics and sensitivity-analysis results are provided in Supplementary Tables S2–S4.

### 2.3 Differential gene expression analysis

Based on thresholds of |log_2_FC| > 0.50 and adjusted P value (P.adj.Val) < 0.05, differentially expressed genes (DEGs) between disease and control samples were identified via the “limma” R package. For visual interpretation, a volcano plot and a clustered heatmap (generated by the “pheatmap” package) were employed to display the differential expression profile.

### 2.4 Weighted gene co-expression network analysis (WGCNA)

Co-expression networks were constructed using the WGCNA R package (Langfelder and Horvath, 2008). Each GEO dataset was preprocessed, normalized, and analyzed independently rather than being merged across platforms. The top 25% most variable genes, ranked by standard deviation, were retained for network analysis. GoodSamplesGenes and hierarchical sample clustering were used for quality control and outlier detection. The soft-thresholding power was selected according to scale-free topology criteria. An adjacency matrix was constructed and transformed into a topological overlap matrix (TOM), followed by hierarchical clustering and dynamic tree cutting to identify gene modules. Module–trait relationships were assessed using module eigengenes, and candidate modules were evaluated based on gene significance (GS) and module membership (MM).

### 2.5 Functional enrichment analysis

Key genes shared between asthma and AD were identified by intersecting the DEGs and genes from the significant WGCNA modules using a Venn diagram. Functional enrichment analysis of these shared genes for Gene Ontology (GO) terms and Kyoto Encyclopedia of Genes and Genomes (KEGG) pathways was conducted with the “clusterProfiler” package. Significantly enriched terms (*P < 0.05*) were visualized using bar plots and scatter plots.

### 2.6 Machine learning screening of key shared genes

Key shared genes were further screened using three machine learning approaches. Least Absolute Shrinkage and Selection Operator (LASSO) regression was carried out with the “glmnet” package. Simultaneously, Support Vector Machine-Recursive Feature Elimination (SVM-RFE) was performed using the “e1071” and “MSVM-RFE” packages. In addition, a Random Forest model incorporating 500 trees was constructed to assess feature importance, with a significance threshold set at 0.9. Genes commonly selected by all three algorithms were defined as the final key shared genes. The diagnostic potential of these genes was evaluated by receiver operating characteristic (ROC) curve analysis within the discovery cohort, implemented with the “pROC” package.

### 2.7 Validation of key shared genes

The predictive performance of the key shared genes was validated in independent external cohorts: two for asthma (GSE147878, GSE69683) and two for AD (GSE5281, GSE63061). ROC curves were constructed, and the area under the curve (AUC) was determined to quantify the classification accuracy.

### 2.8 Mouse model of asthma

Female BALB/c mice (6 weeks old) were obtained from the Animal Experimental Center of Wuhan University. An ovalbumin (OVA)-induced asthma model was established. Briefly, mice were sensitized via intraperitoneal injection of 20 μg OVA (Sigma) emulsified in 2 mg Imject™ Alum (Thermo Scientific) on day 0 and day 14. From days 21–23, the mice received an intranasal challenge with 100 μg OVA under anesthesia induced by 1% pentobarbital sodium (60–80 mg/kg) (Lin et al., 2023; Deng et al., 2020). All mice were humanely euthanized by CO_2_ asphyxiation 24 h after the final challenge. For molecular validation, three animals were included in each control and asthma group. Animals were allocated by simple randomization, and the sample size was based on previous studies using comparable experimental designs; no formal *a priori* power calculation was performed. Blinding was not performed, and no animals or data points were excluded from the analyses. CD163 and CHST6 mRNA and protein expression were assessed as exploratory molecular outcomes. The study was approved by the Wuhan University Ethics Committee (Approval No. WP20230530) and conducted in compliance with the ARRIVE guidelines.

### 2.9 Mouse model of AD

Female 3xTg AD mice on a C57BL/6 background were kindly provided by the Yaozhaohui Laboratory. All animals were maintained under specific pathogen-free (SPF) conditions at a constant temperature of 24 °C ± 1.5 °C, with free access to food and water, as previously described (Wu et al., 2024). For molecular validation, three animals were included in each control and disease group. Animals were allocated by simple randomization, and the sample size was based on previous studies using comparable experimental designs; no formal *a priori* power calculation was performed. Blinding was not performed. No animals or data points were excluded from the analyses. CD163 and CHST6 mRNA and protein expression were assessed as exploratory molecular outcomes. Only female animals were used.

### 2.10 Western blot

Lung and brain tissue proteins were separated by 10% SDS-PAGE and transferred to a 0.45 μm PVDF membrane. After blocking with 5% skim milk, the membrane was incubated overnight at 4 °C with primary antibodies against β-Tubulin (Servicebio, 60004-1-Ig), β-actin (HUABIO, HA722023), CHST6 (Novus, NBP2-92007), and CD163 (Proteintech, 16646-1-AP) at a dilution of 1:1000. After washing, membranes were incubated with HRP-conjugated secondary antibodies (Abclonal, 1:2000). Protein bands were visualized using an enhanced chemiluminescence (ECL) kit (HYCEZMBIO, HYC0316) and captured using a chemiluminescence imaging system (Tanon 5200) with automatic exposure mode. Band intensities were quantified using ImageJ software. Target protein expression was normalized to the corresponding loading control from the same lane (β-tubulin for asthma samples and β-actin for AD samples). Full uncropped blot images are provided in the Supplementary Material.

### 2.11 RT-PCR

Total RNA was isolated from lung and hippocampal tissues using TRIzol reagent (Servicebio, G3013). cDNA was synthesized using a gDNA removal kit (TIANGEN, KR118), and quantitative PCR was performed using SYBR Green mix (CWBIO, CW0956M). GAPDH was used as the reference gene. Three biological replicates were analyzed per group, and each sample was measured in three technical replicates. The same animals were used for RT-qPCR and Western blot analyses. Relative gene expression was calculated using the 2^−ΔΔCt^ method. Melt-curve analysis was performed after amplification to monitor amplification specificity. Numerical primer-efficiency values were unavailable from the archived experimental records and were therefore not reported. Primer sequences, including those for GAPDH, are provided in Table 2.

**TABLE 2:** Primer sequences used for RT-PCR.

Primer (mouse)	Sequence (5′to 3′)
CD163-F	CGG​CTG​CCT​CCA​CCT​CTA​AGT
CD163-R	ATG​AAG​ATG​CTG​GCG​TGA​CA
CHST6-F	GAC​AGC​CTC​TAC​TAC​TTC​CGA​G
CHST6-R	CAG​AGA​CTC​TGG​TCT​TTC​GGC​A
GAPDH-F	AGG​TCG​GTG​TGA​ACG​GAT​TTG
GAPDH-R	TGT​AGA​CCA​TGT​AGT​TGA​GGT​CA

### 2.12 SHAP analysis

Five machine learning algorithms—Partial Least Squares (PLS), Random Forest (RF), Logistic Regression (LR), XGBoost, and Neural Network—were implemented to validate the diagnostic hub genes. Model performance was evaluated using ROC curves and AUC values to identify the optimal classifier. To interpret the best-performing model, SHAP (SHapley Additive exPlanations) analysis was employed. SHAP bar plots, summary (beeswarm) plots, and dependence plots were generated to visualize global feature importance, the directional impact of gene expression on AD risk, and specific feature-risk correlations, respectively.

### 2.13 Development and validation of a binary LR model

A binary logistic regression (LR) model was implemented to estimate the regression coefficients of the hub genes (CD163 and CHST6) and generate individualized risk scores for each patient. To facilitate model interpretation, the “rms” R package was utilized to develop a nomogram incorporating these key molecular features. The model was evaluated using calibration curve analysis, decision curve analysis (DCA), and ROC curve metrics. External validation of the diagnostic model was performed using the independent GSE5281 dataset.

### 2.14 Gene set enrichment analysis (GSEA)

To elucidate the functional profiles of the key shared genes, GSEA (Subramanian et al., 2007) was conducted using the “clusterProfiler” R package. This analysis aimed to identify significantly enriched GO terms and KEGG pathways within the asthma and AD groups, with a nominal P value of less than 0.05 defined as the cutoff for significance.

### 2.15 Immune-cell signature analysis

Expression matrices were log2-transformed when required and normalized using the normalizeBetweenArrays function in the limma R package. Enrichment scores for 28 immune-cell signatures were estimated by ssGSEA using the GSVA R package and the immune. gmt gene-set collection (Charoentong et al., 2017). ssGSEA was performed with method = “ssgsea”, kcdf = “Gaussian”, and abs. ranking = TRUE, followed by min–max scaling of enrichment scores to 0–1. Disease–control comparisons were performed separately within each dataset, without pooling samples across different tissue types. Spearman correlation analysis was used to assess associations between CD163 or CHST6 expression and immune-cell signature scores within disease samples. All correlation analyses were performed independently within each dataset. P values were adjusted for multiple testing using the Benjamini–Hochberg method.

### 2.16 Single-cell RNA sequencing analysis

Single-cell/single-nucleus RNA-sequencing data were obtained from GSE243292, GSE172495, and GSE145013. GSE243292 included 15 donors classified by AT(N) status: A^−^T^−^ normal controls (*n* = 2), A^+^T^−^ pathological-aging donors (*n* = 5), and A^+^T^+^ AD donors (*n* = 8). All donors were retained for visualization and cell-type annotation; primary disease–control comparisons were performed between A^+^T^+^ and A^−^T^−^ donors, with the A^+^T^−^ group analyzed separately. GSE172495 comprised PBMCs from five patients with severe asthma and three healthy controls under unstimulated and Poly I:C conditions; disease–control comparisons used the unstimulated samples. GSE145013 comprised IL-13- and BSA-treated airway epithelial cultures from two donors and was analyzed as an *in vitro* asthma-related epithelial model. Cells/nuclei with 300–4,500 detected genes and <10% mitochondrial transcripts were retained. Data were processed using Seurat, with Harmony applied for batch correction using sample/dataset of origin as the batch variable. Shared-nearest-neighbor clustering was performed at a resolution of 0.5, followed by UMAP visualization. Cell types were annotated using canonical markers, with the top five markers for each cell type provided in Supplementary Table S7. CD163 and CHST6 were quantified by mean expression and percentage of expressing cells within each cell type and sample. Donor-level pseudobulk analyses were used for disease–control comparisons to avoid cell-level pseudoreplication. Because GSE145013 contained only two donors, IL-13 versus BSA comparisons were considered exploratory cell-level analyses. Detailed dataset composition and analysis parameters are provided in Supplementary Table S6.

### 2.17 Statistical analysis

All statistical computations and data analyses were carried out using R software (version 4.2.3). For comparisons between two groups, the appropriate statistical test (Student’s t-test or Wilcoxon test) was selected based on data distribution. Correlation analyses were performed using either Pearson or Spearman methods. A P value below 0.05 was considered indicative of statistical significance.

## 3 Results

### 3.1 Mendelian Randomization analysis of asthma on AD risk

Using 16 independent genetic instruments, two-sample MR analysis suggested a nominal positive association between genetic liability to childhood asthma and AD risk (IVW: OR = 1.063, 95% CI: 1.000–1.129, *P* = 0.049). However, this association did not remain statistically significant after FDR correction (FDR-adjusted P = 0.653). All genetic instruments had F-statistics >10, indicating a low likelihood of weak-instrument bias. Cochran’s Q and MR-Egger intercept tests showed no evidence of significant heterogeneity or directional horizontal pleiotropy, respectively, and leave-one-out analysis showed that the direction of the association remained positive after sequential exclusion of individual SNPs, although nominal statistical significance was not retained in all iterations (Figure 2; Supplementary Tables S2–S4). Overall, these findings suggest a potential genetic association between childhood asthma liability and AD risk and should be interpreted cautiously.

**FIGURE 2:**
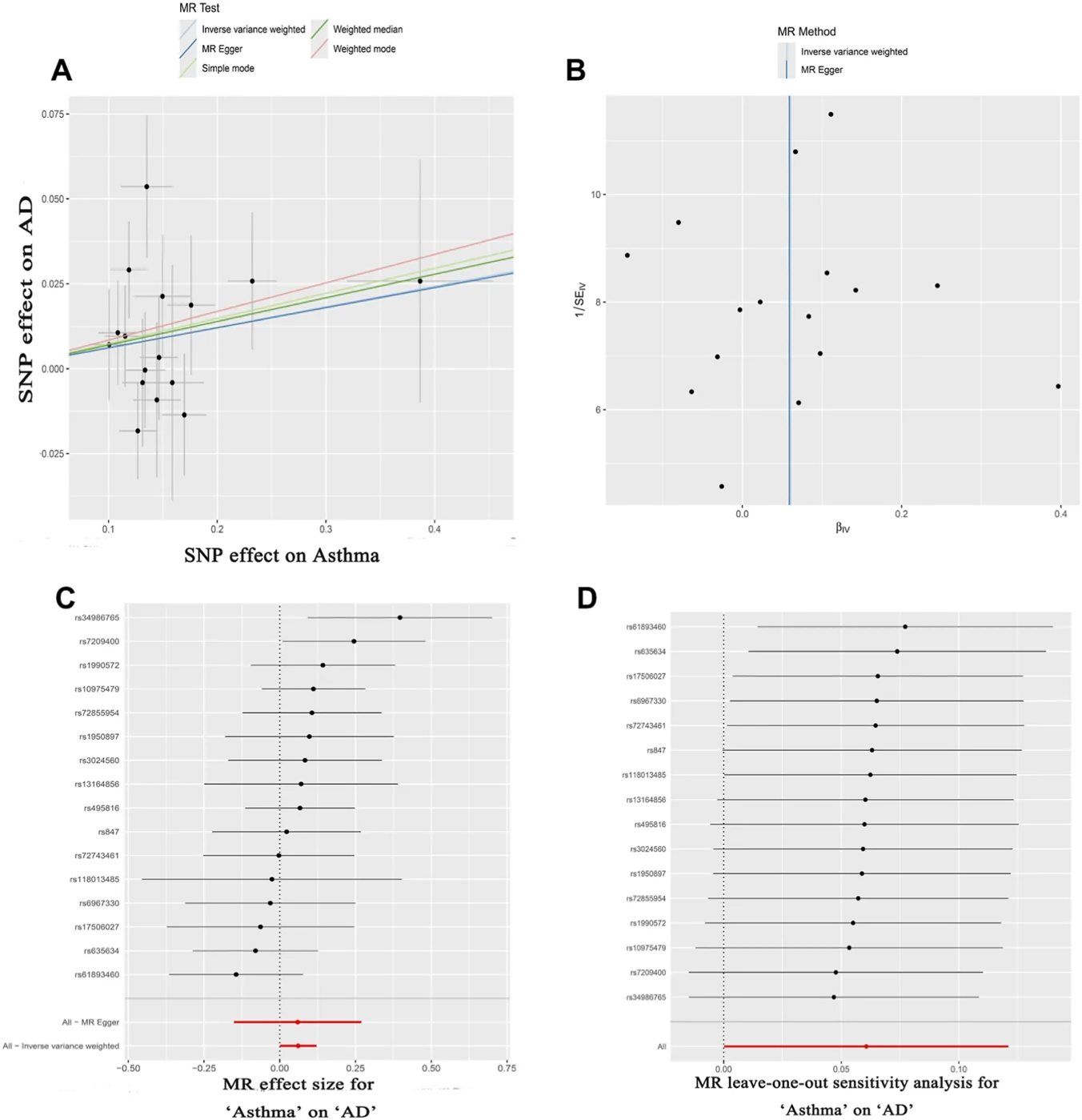
Mendelian randomization analysis results. **(A)** Scatter plot showing the associations of individual genetic instruments with childhood asthma and AD. Lines represent estimates obtained using IVW, MR-Egger, weighted median, simple mode, and weighted mode methods. **(B)** Funnel plot for visual assessment of the symmetry of individual SNP estimates. **(C)** Forest plot showing individual SNP estimates and pooled MR estimates. **(D)** Leave-one-out sensitivity analysis evaluating the influence of individual SNPs on the overall IVW estimate. Exposure: childhood asthma (age <16; FinnGen R12, finngen_R12_ASTHMA_CHILD_EXMORE); outcome: AD (ieu-b-2).

### 3.2 Identification of differentially expressed genes

To identify genes specifically associated with asthma and AD, we analyzed two gene expression datasets: GSE143303 (from endobronchial biopsy tissue) for asthma and GSE29378 (from brain tissue) for AD. In the asthma dataset GSE143303, we identified 104 upregulated and 104 downregulated DEGs (Figures 3A,B). In the AD dataset GSE29378, we found 19 upregulated and 94 downregulated DEGs (Figures 3C,D).

**FIGURE 3:**
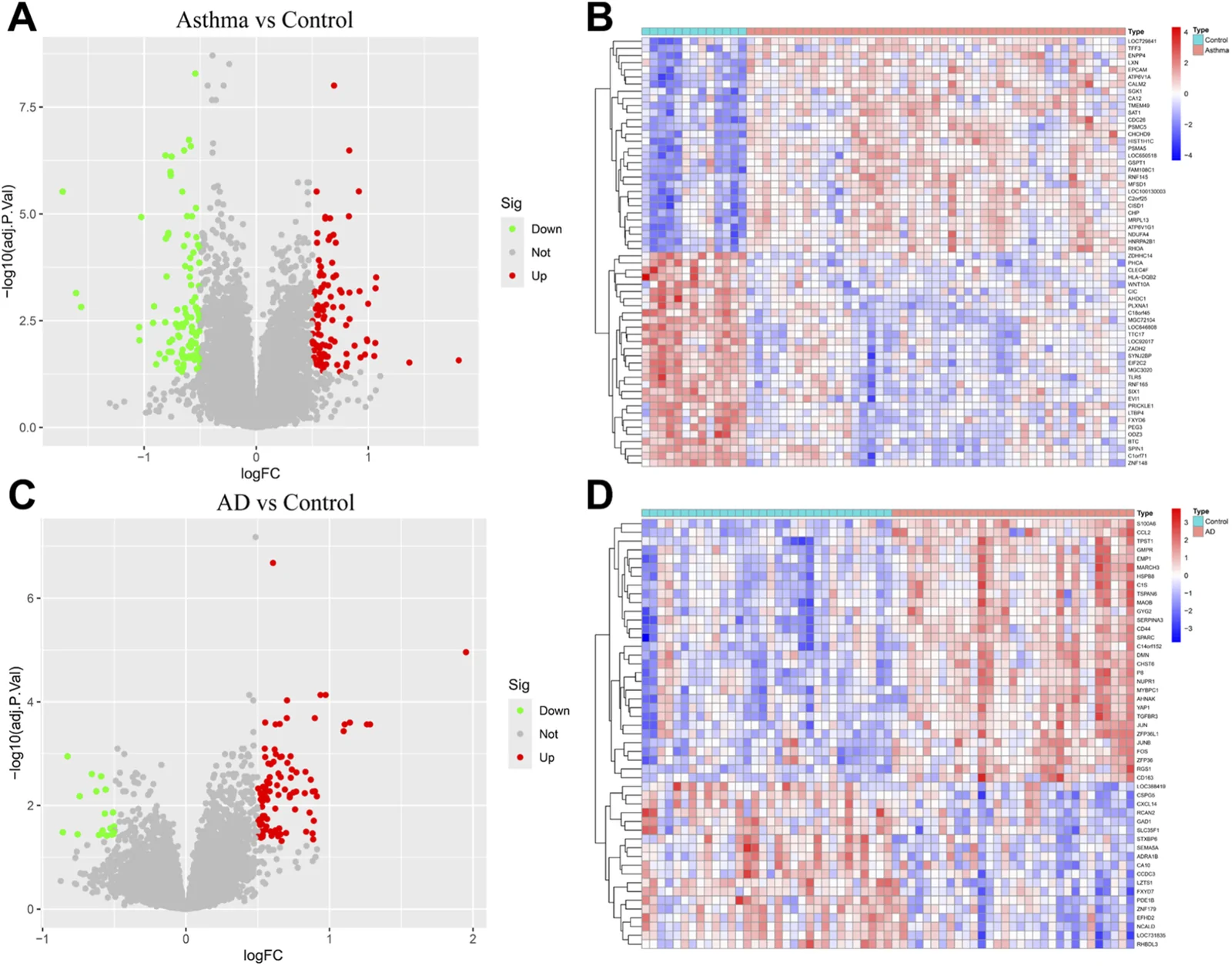
Identification of Differentially Expressed Genes in Asthma and AD. **(A)** Asthma DEG volcano plot. **(B)** Asthma DEG heatmap. **(C)** AD DEG volcano plot. **(D)** AD DEG heatmap. Criteria: |log_2_FC| > 0.5, adjusted P value <0.05.

### 3.3 Identification of key modules in asthma and AD by WGCNA

Employing WGCNA, we built a scale-free co-expression network by applying a soft-thresholding power that achieved a scale-free fit index R^2^ of 0.9 (Figures 4A,B). This process defined six key modules in each disease after merging highly correlated gene clusters (Figures 4C,D). Correlation analysis with clinical traits highlighted the red module (633 genes) as most strongly linked to asthma (r = 0.62, *P* = 1 × 10^−7^), and the yellow module (624 genes) as the top candidate associated with AD (r = 0.42, *P* = 7 × 10^−4^; Figures 4E,F).

**FIGURE 4:**
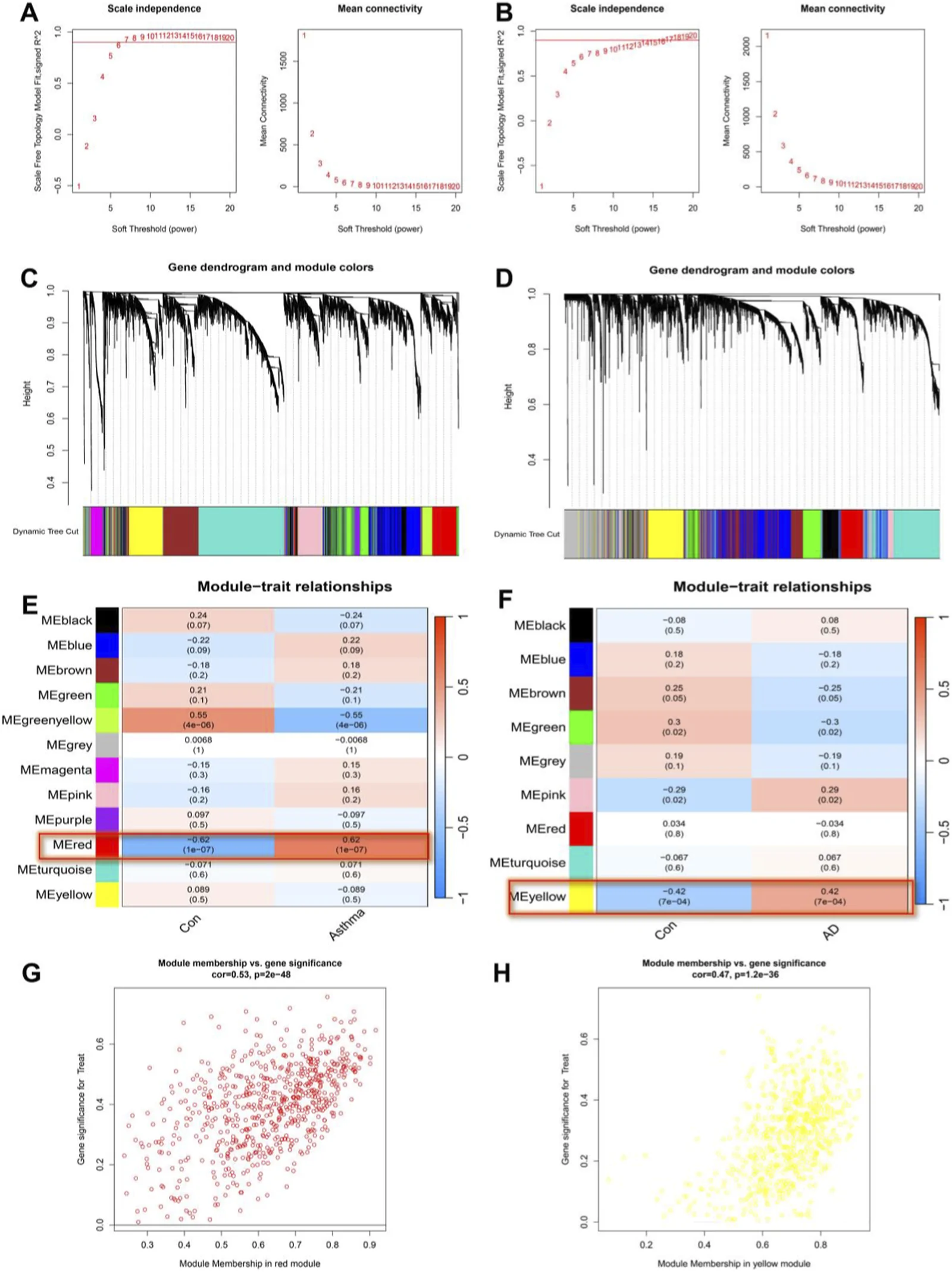
WGCNA identifies key modules associated with asthma and AD. **(A,B)** Soft-thresholding power selection for the asthma and AD datasets, respectively. **(C,D)** Gene clustering dendrograms and module assignments for asthma and AD. **(E,F)** Heatmaps of module-trait correlations. **(G)** Correlation of GS for asthma with MM in the asthma-related red module. **(H)** Correlation of GS for AD with MM in the AD-related yellow module.

Significant positive correlations between GS and MM were found for both key modules (red: cor = 0.53, *P* = 2e^−48^; yellow: cor = 0.47, *P* = 1.2e-36) (Figures 4G,H). Genes from these two modules were subsequently analyzed to explore the potential mechanisms linking asthma and AD.

### 3.4 Analysis of shared genes and functional enrichment

We identified candidate genes shared between asthma and AD from two sources. The intersection of asthma-related and AD-related DEGs yielded five genes: CD163, CHST6, DUSP1, AKRIC3, and C11orf70 (Figure 5A). Separately, the intersection of disease-associated gene modules from the WGCNA analysis yielded 19 additional genes, including ZNF800, LOC649049, CPNE3 (Figure 5B). The combined set of 24 genes (Figure 5C) was subjected to functional enrichment analysis.

**FIGURE 5:**
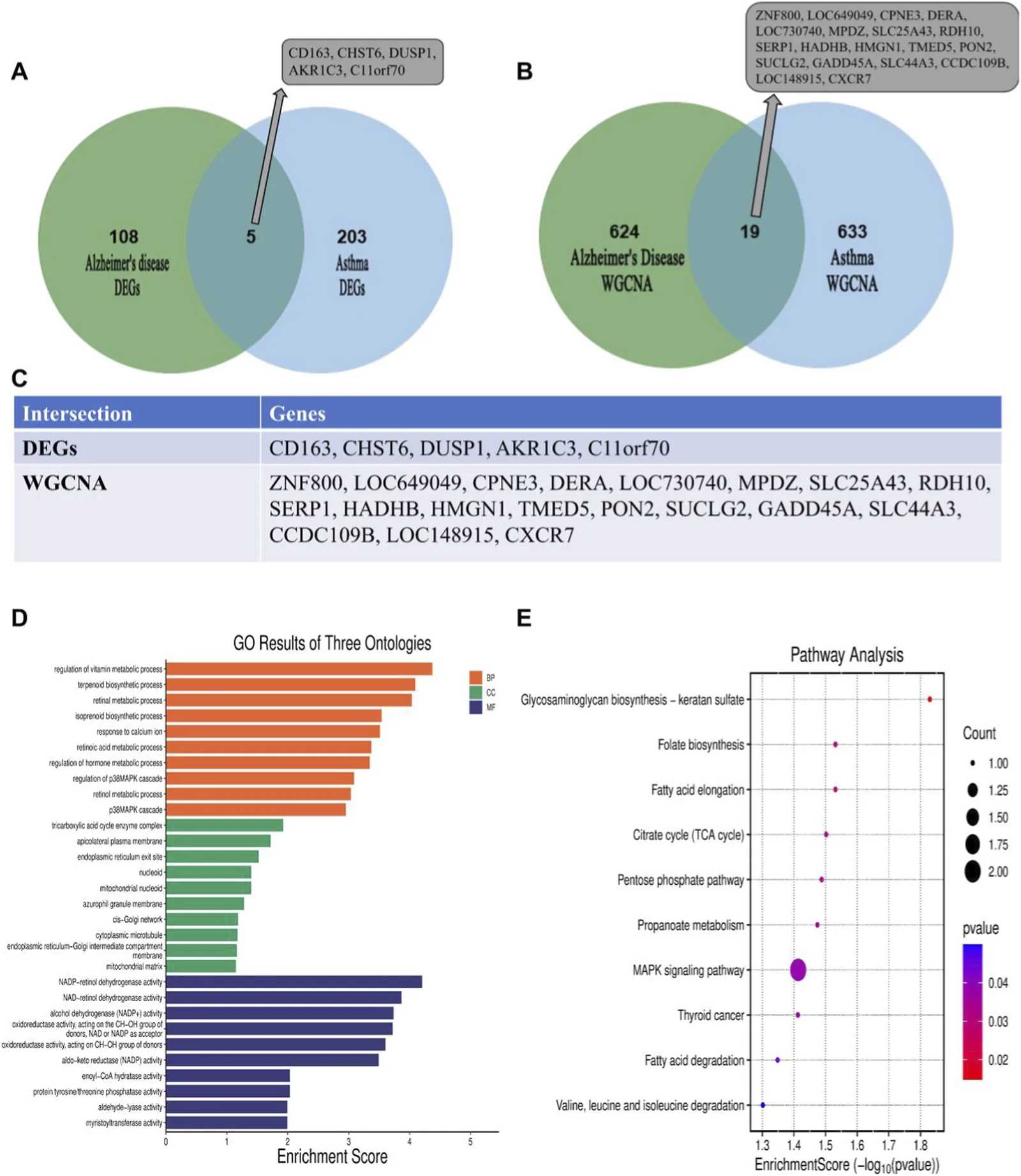
Identification and functional analysis of shared genes between asthma and AD. **(A)** Venn diagram of the overlap between asthma-related and AD-related DEGs. **(B)** Venn diagram of the overlap between asthma-associated and AD-associated WGCNA modules. **(C)** Table listing the 24 candidate shared genes. **(D)** Significantly enriched Gene Ontology (GO) terms. **(E)** Significantly enriched KEGG pathways.

GO analysis revealed significant enrichment for biological processes (BP) including the regulation of vitamin metabolic process and terpenoid biosynthetic process. For cellular components (CC), significant terms included the tricarboxylic acid cycle enzyme complex and the apicolateral plasma membrane. The most enriched molecular function (MF) term was NAD(P)^+^-dependent oxidoreductase activity. KEGG pathway analysis showed significant enrichment in the MAPK signaling pathway and pathways related to energy metabolism (Figures 5D,E).

### 3.5 Identification of potential shared diagnostic genes by machine learning algorithms

For asthma, LASSO (Figures 6A,B), RF (Figures 6C,G), and SVM-RFE (Figures 6D,E) were applied independently, and the intersection of the three algorithms identified CD163 and CHST6 as hub genes (Figure 6F). The diagnostic performance based on these genes was excellent, with AUC values reaching 0.979 for the LASSO model, 0.835 for SVM-RFE, and 0.795 for RF (Supplementary Figures S1A,D,H).

**FIGURE 6:**
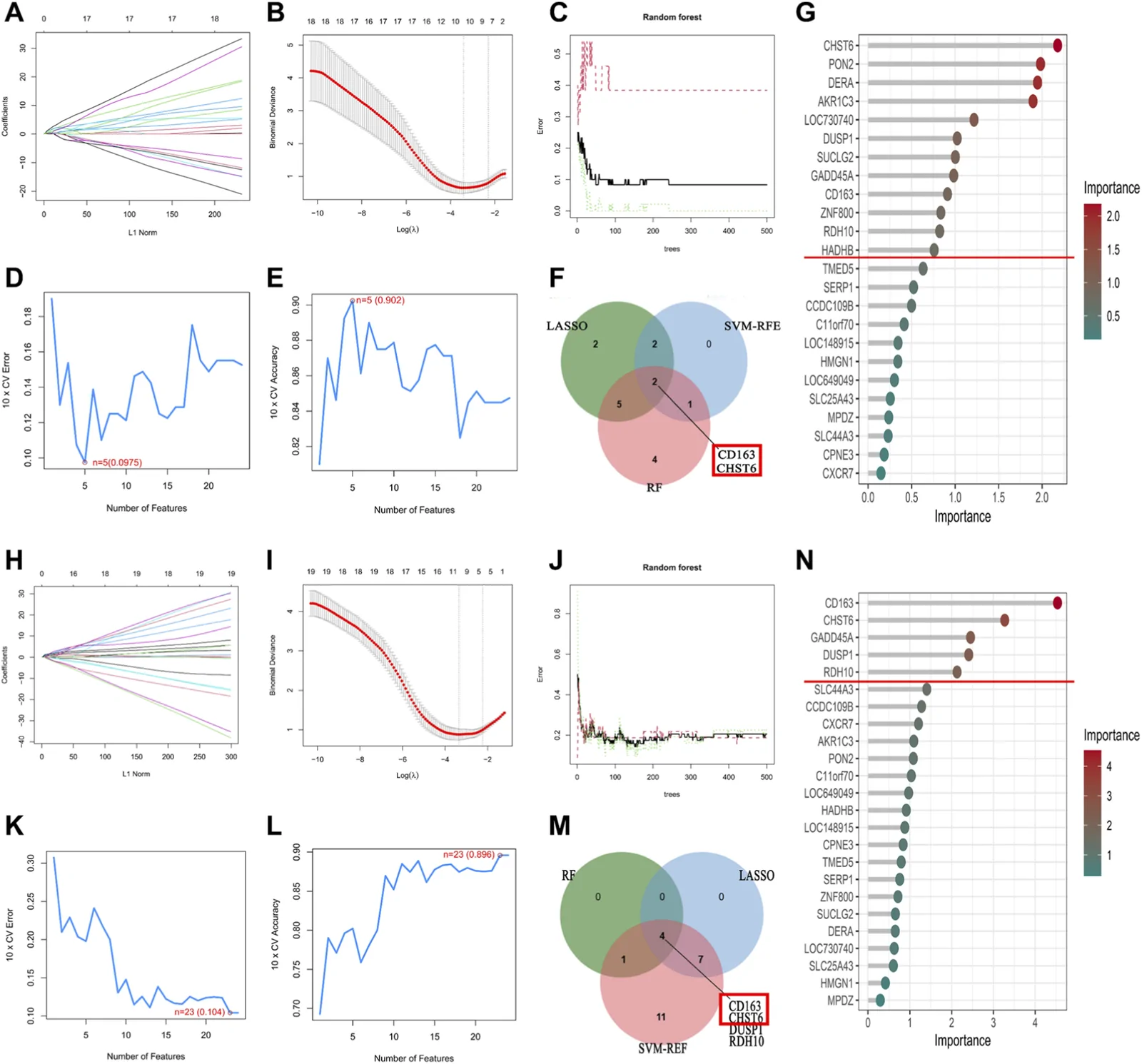
Screening of candidate diagnostic genes using machine learning algorithms. **(A,B)** LASSO coefficient profile and cross-validation curve for asthma. **(C)** RF error rate versus number of trees for asthma. **(D,E)** SVM-RFE cross-validation error and accuracy curves for asthma. **(F)** Venn diagram of hub genes identified by the three algorithms for asthma. **(G)** Variable importance of the top 12 genes from the RF model for asthma. **(H,I)** LASSO coefficient profile and cross-validation curve for AD. **(J)** RF error rate versus number of trees for AD. **(K,L)** SVM-RFE cross-validation error and accuracy curves for AD. **(M)** Venn diagram of hub genes identified by the three algorithms for AD. **(N)** Variable importance of the top 5 genes from the RF model for AD.

For AD, LASSO (Figures 6H,I), RF (Figures 6J,N), and SVM-RFE (Figures 6K,L) were similarly applied, and their consensus identified four hub genes: CD163, CHST6, DUSP1, and RDH10 (Figure 6M). These genes also demonstrated strong diagnostic capability, with AUC values of 0.969 for the LASSO model, 0.800 for SVM-RFE, and 0.882 for RF (Supplementary Figures S1B,E,I).

### 3.6 Expression analysis of CD163 and CHST6

We further evaluated the expression and diagnostic efficacy of CD163 and CHST6 in the training sets. In the asthma cohort (GSE143303), both genes showed notable upregulation compared with controls (*P* < 0.001; Figure 7A) and exhibited high diagnostic value, with area under the curve (AUC) values of 0.815 for CD163 and 0.838 for CHST6 (Figure 7B). Consistent results were observed in the AD cohort (GSE29378), where CD163 and CHST6 were also significantly elevated (*P* < 0.001; Figure 7C) and demonstrated strong classification ability, achieving AUCs of 0.855 and 0.812, respectively (Figure 7D).

**FIGURE 7:**
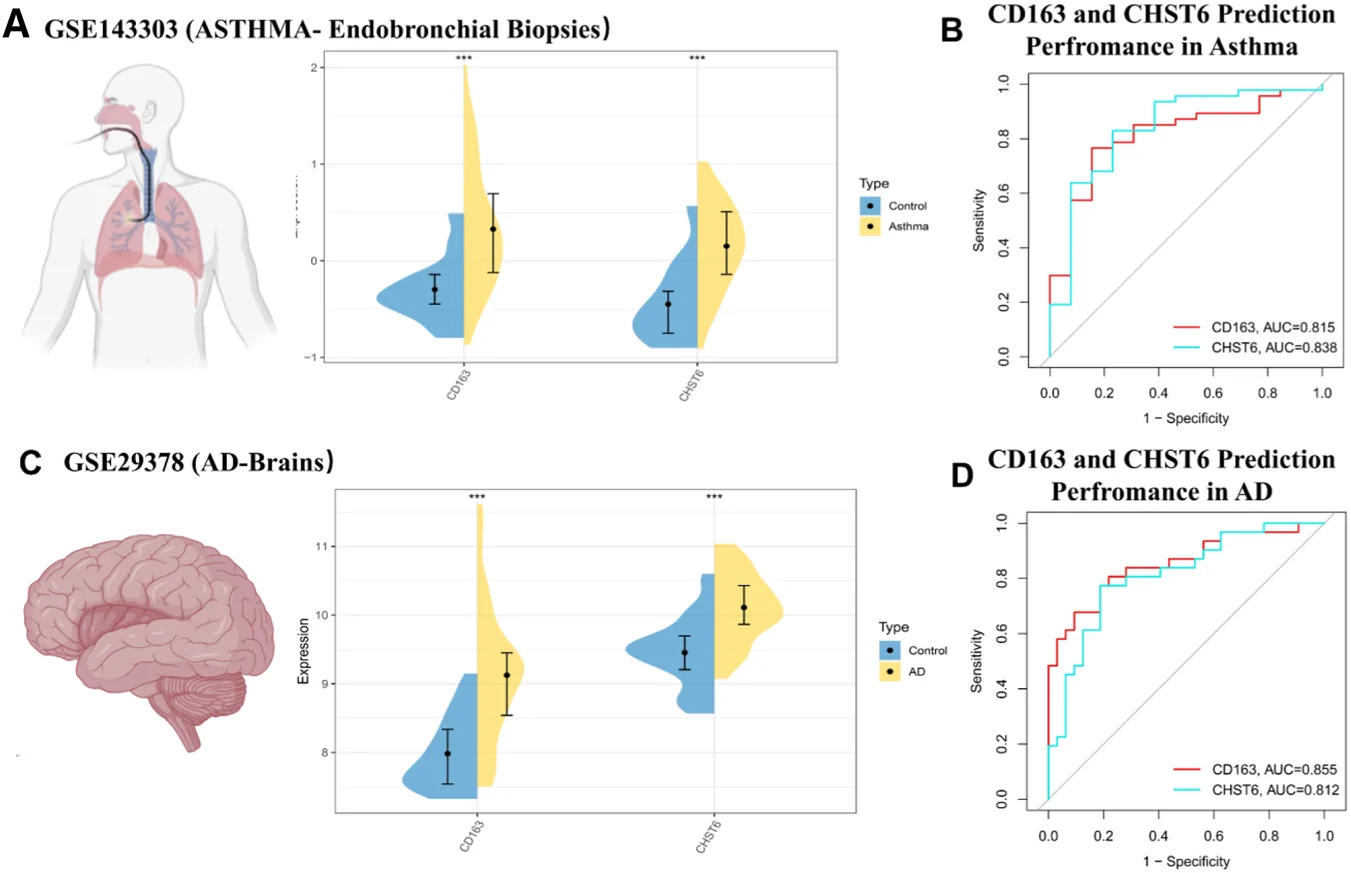
Expression and Diagnostic Value of CD163 and CHST6 in Training Datasets. **(A)** Expression levels of CD163 and CHST6 in the asthma training dataset (GSE143303; endobronchial biopsies; mean ± SEM). **(B)** ROC curves assessing the diagnostic power of CD163 and CHST6 in asthma. **(C)** Expression levels of CD163 and CHST6 in the AD training dataset (GSE29378; brain tissue; mean ± SEM). **(D)** ROC curves assessing the diagnostic power of CD163 and CHST6 in AD. *P < 0.05, **P < 0.01, ***P < 0.001. Schematic elements were created with BioRender.com.

### 3.7 Multi-cohort external validation of CD163 and CHST6

To further validate our findings, we assessed the diagnostic performance of CD163 and CHST6 in independent external datasets. This included endobronchial biopsies (GSE147878) and peripheral blood (GSE69683) for asthma, as well as brain tissue (GSE5281) and peripheral blood (GSE63061) for AD. Consistent with the training results, both genes remained significantly upregulated in the external asthma endobronchial biopsy dataset (*P* < 0.001) and exhibited good diagnostic performance, with AUCs of 0.767 for CD163 and 0.807 for CHST6 (Figures 8A,B). Similarly, in the external AD brain tissue dataset, significant upregulation of both genes in these central tissues (*P* < 0.001) was confirmed, with AUCs of 0.678 for CD163 and 0.756 for CHST6 (Figures 8C,D).

**FIGURE 8:**
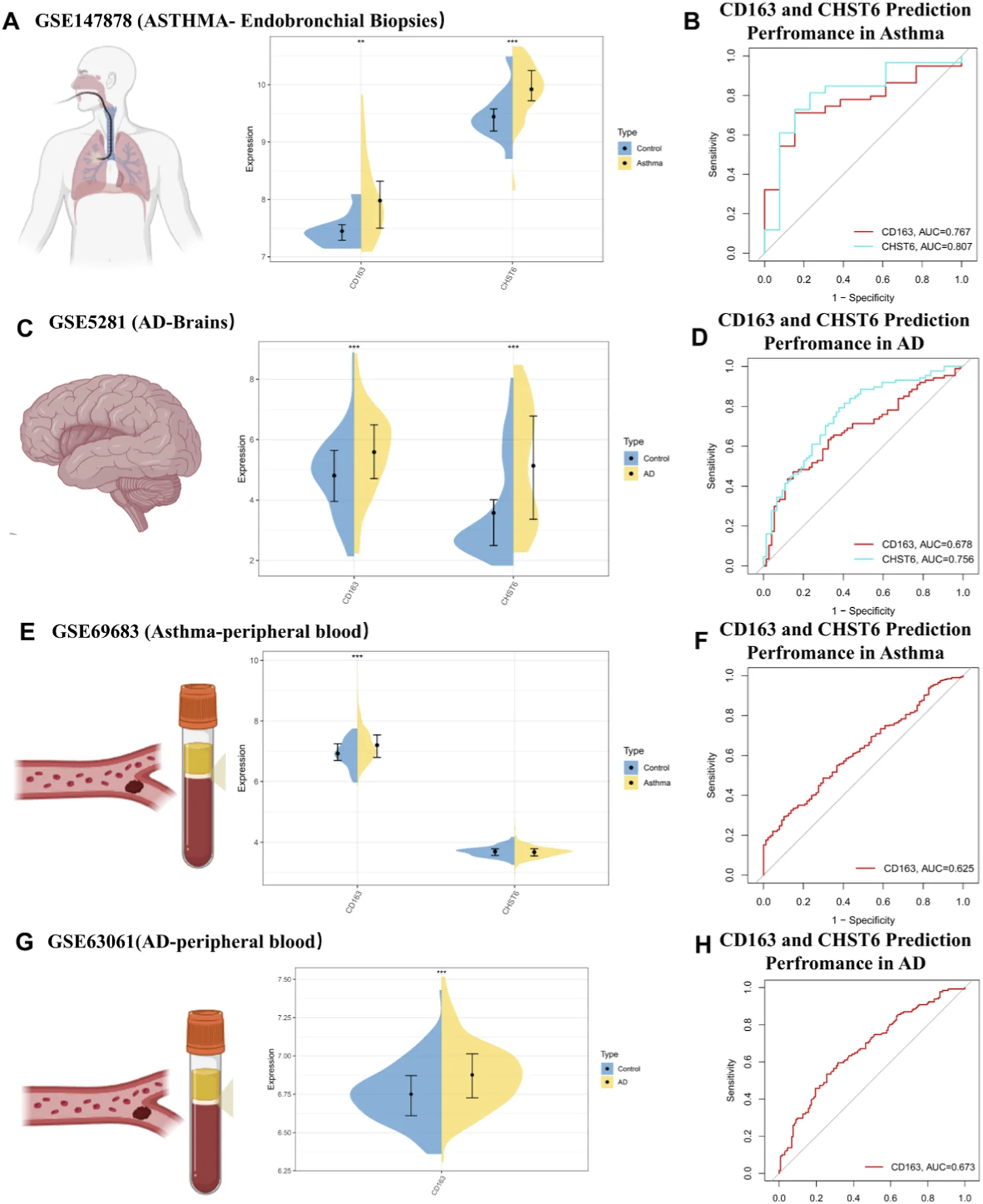
Multi-Tissue External Validation of CD163 and CHST6 in Independent Asthma and AD Cohorts. **(A,B)** CD163 and CHST6 expression with ROC analysis in an external asthma endobronchial biopsy cohort (GSE147878). **(C,D)** Gene expression and diagnostic ROC evaluation in an external AD brain tissue cohort (GSE5281). **(E,F)** Validation in an external asthma peripheral blood cohort (GSE69683). **(G,H)** Validation in an external AD peripheral blood cohort (GSE63061). *P < 0.05, **P < 0.01, ***P < 0.001. Schematic elements were created with BioRender.com.

We next explored their potential in peripherally accessible blood. In the asthma blood dataset, CD163 levels were elevated (*P* < 0.001) with an AUC of 0.625 (Figures 8E,F). In the AD blood dataset, CD163 was also upregulated (*P* < 0.001) with an AUC of 0.673 (Figures 8G,H). Due to its absence from the transcriptomic panels of these specific blood datasets, CHST6 could not be evaluated.

### 3.8 MR analysis suggests genetic associations of CD163 with asthma- and AD-related phenotypes

MR analyses showed directionally consistent nominal positive associations of genetically predicted CD163 expression and plasma protein levels with AD- and asthma-related phenotypes. Genetically predicted CD163 expression was associated with Alzheimer disease (IVW: OR = 1.209, 95% CI: 1.036–1.410, *P* = 0.016) and the asthma-and-allergy phenotype (OR = 1.210, 95% CI: 1.036–1.413, *P* = 0.016), whereas genetically predicted plasma CD163 levels were associated with dementia in Alzheimer disease (OR = 1.079, 95% CI: 1.005–1.158, *P* = 0.035) and allergic asthma (OR = 1.052, 95% CI: 1.008–1.097, *P* = 0.019) (Figures 9A–D). However, none of the primary IVW associations remained statistically significant after FDR correction (FDR-adjusted *P* = 0.209, 0.466, 0.390, and 0.276, respectively).

**FIGURE 9:**
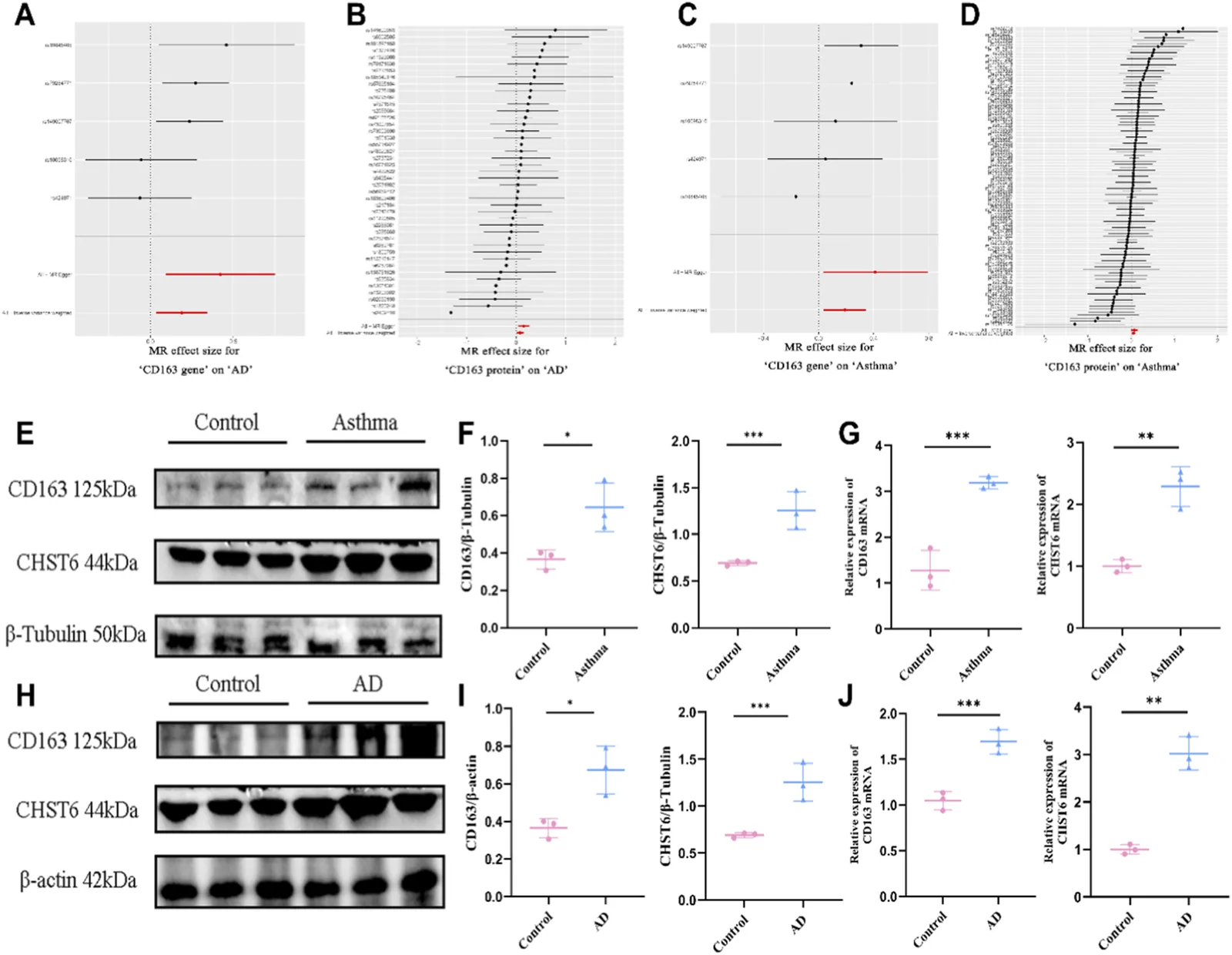
Multi-dimensional validation of CD163 and CHST6 via Mendelian Randomization and *in vivo* experiments. **(A–D)** Forest plots of MR estimates for genetically predicted CD163 expression and plasma protein levels. **(A)** CD163 gene expression and Alzheimer disease. **(B)** Plasma CD163 levels and dementia in Alzheimer disease. **(C)** CD163 gene expression and the asthma-and-allergy phenotype. **(D)** Plasma CD163 levels and allergic asthma. Red lines indicate pooled estimates obtained using the inverse variance weighted (IVW) method. **(E)** Western blot analysis of CD163 and CHST6 expression in lung tissues of female control and asthma mice. **(F)** Quantitative analysis of protein levels in the asthma model (*n* = 3 biological replicates per group). **(G)** RT-qPCR analysis of mRNA expression in the asthma model (*n* = 3). **(H)** Western blot analysis in hippocampal tissues of female control and AD mice. **(I)** Quantitative analysis of protein levels in the AD model (*n* = 3). **(J)** RT-qPCR analysis of mRNA expression in the AD model (*n* = 3 biological replicates per group). Data are presented as mean ± SEM. *P < 0.05, **P < 0.01, ***P < 0.001.

For the CD163 expression analyses, no significant heterogeneity was detected for Alzheimer disease (Q = 7.07, *P* = 0.132) or asthma and allergy (Q = 3.05, *P* = 0.549), and the corresponding MR-Egger intercept tests were also non-significant (*P* = 0.227 and *P* = 0.299, respectively). No significant heterogeneity was detected for the CD163 expression–Alzheimer disease, CD163 expression–asthma-and-allergy, or plasma CD163–dementia in Alzheimer disease analyses, whereas significant heterogeneity was observed for the plasma CD163–allergic asthma analysis. Leave-one-out analyses generally showed consistent directions of effect after sequential exclusion of individual SNPs (Supplementary Figure S2). Detailed instrument characteristics, MR estimates, and sensitivity analyses are provided in Supplementary Tables S2–S4. CHST6 was not evaluated by MR because insufficient genetic instruments met the prespecified genome-wide significance threshold.

### 3.9 Independent experimental validation of CD163 and CHST6 in mouse models

RT-qPCR and Western blot analyses showed significantly increased mRNA and protein levels of CD163 and CHST6 in lung tissues from asthmatic mice and hippocampal tissues from AD mice compared with their respective controls (Figures 9E–J).

### 3.10 SHAP explanatory analysis

Five machine learning algorithms, namely, PLS, RF, Logistic Regression, XGBoost, and Neural Network, were implemented to validate the diagnostic robustness of the identified hub genes (Figure 10A). Among these, the PLS model exhibited the best overall predictive performance, as evidenced by the highest AUC value (0.869). To understand the underlying mechanisms of the PLS model, SHAP values were calculated to quantify the contribution size of each feature. The barplot analysis (Figure 10B) and SHAP summary plots (Figure 10C) further highlighted CD163 as the key determinant for sample classification, indicating that both genes (CD163 and CHST6) had positive impacts on the prediction outcomes. These findings suggested that elevated expression levels of these genes were associated with the disease state, contrasting with the expression patterns in control samples. Scatter plots (Figure 10D) confirmed positive correlations between SHAP values and the expression levels of the two genes, suggesting that both genes contributed to sample classification.

**FIGURE 10:**
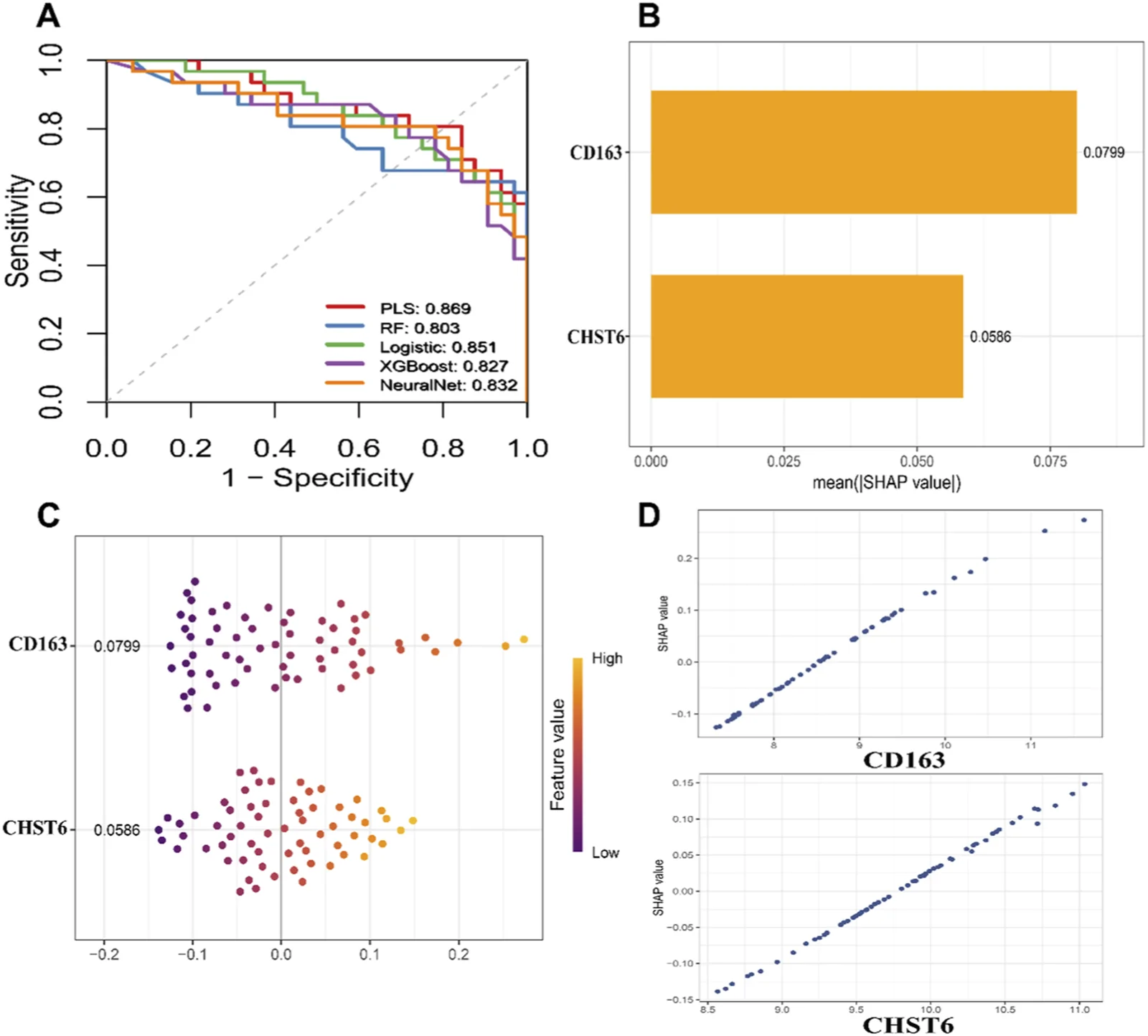
Construction of the AD classification model and SHAP explanatory analysis. **(A)** ROC curves comparing five machine learning algorithms. **(B)** Bar plot ranking the feature importance based on mean absolute SHAP values in the PLS model. **(C)** SHAP summary plot (Beeswarm plot). Each dot represents a sample; red color indicates high gene expression, which corresponds to positive SHAP values (higher probability of AD classification). **(D)** SHAP dependence plots showing the positive correlation between gene expression levels and AD risk contribution.

### 3.11 Construction and validation of the LR diagnostic model

The diagnostic model was constructed using the hub genes CD163 and CHST6, with the final risk score calculated as Risk Score = −30.538 + (1.674 × CHST6) + (1.678 × CD163). Patients in the disease group had substantially higher risk scores compared to healthy controls (Figure 11A). A risk nomogram for AD was devised based on the expression levels of these hub genes, aiming to formulate a more clinically relevant classification model (Figure 11B). By constructing clinical calibration curves (Figure 11C) and decision curves (Figure 11D), it became evident that the proposed model possessed high predictive capabilities for AD. The model achieved an AUC of 0.879 in the training set (Figure 11E). The sensitivity and specificity curves (Figure 11F) collectively demonstrated that the model exhibits a high level of classification performance.

**FIGURE 11:**
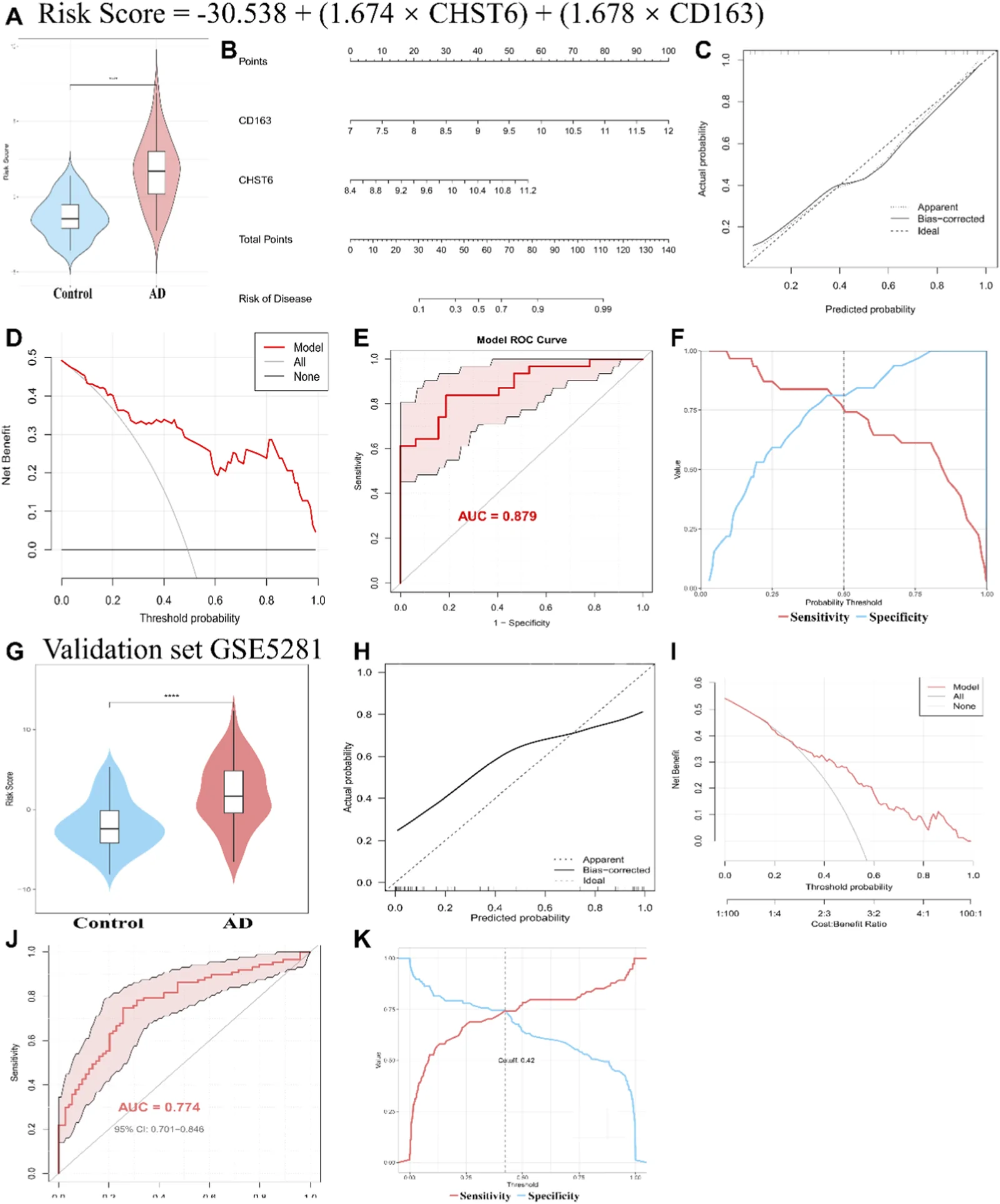
Hub gene-based classification model and diagnostic performance in the training and external validation sets. **(A)** The risk scores calculated by the model for patients with AD and healthy controls. **(B)** Nomogram constructed to predict the risk of disease. **(C)** Calibration curves. **(D)** DCA evaluating the prediction efficacy and decision-curve performance of the model. **(E)** ROC evaluating the prediction efficacy. **(F)** Sensitivity and specificity curves. **(G–K)** External validation in the GSE5281 dataset **(G)** Risk score distribution between Control and AD groups. **(H)** Calibration curve. **(I)** DCA curve. **(J)** ROC curve. **(K)** Sensitivity and specificity curves.

For external validation in the GSE5281 dataset, risk scores were similarly elevated in AD patients (Figure 11G). Both the calibration curve (Figure 11H) and DCA (Figure 11I) further confirmed the model’s reliability and model performance in an independent cohort. Notably, the model maintained a satisfactory AUC of 0.774 (Figure 11J), with an optimal cutoff value of 0.42 identified by sensitivity and specificity analysis (Figure 11K). These findings substantiate the retained discrimination in the external cohort of our diagnostic signature for AD.

### 3.12 Single-gene GSEA of the shared diagnostic genes

To further explore the associated functional pathways of the hub genes in diseases, we performed a single-gene GSEA (Supplementary Figure S3). In asthma, both genes were primarily enriched in immune-related pathways, specifically T cell activation and cytokine interactions for CD163, and cilia assembly for CHST6. Conversely, in AD, the functional profile shifted toward neuronal processes. Both genes were co-enriched in neurotransmitter transport and synaptic functions, while KEGG analysis highlighted pathways related to neurodegenerative diseases and JAK-STAT signaling.

### 3.13 Immune-cell signature analysis

ssGSEA identified differential immune-cell signature enrichment patterns between disease and control samples within the asthma and AD datasets (Figures 12A,B). Within disease samples, CD163 and CHST6 expression showed tissue-dependent associations with immune-cell signatures. In asthma, CD163 showed a positive association with the MDSC signature, whereas CHST6 showed a negative association with the natural killer T-cell signature. In AD, CD163 and CHST6 showed associations with plasmacytoid dendritic-cell-related signatures and other immune-cell signatures (Figures 12C–F). Statistical significance was assessed using Benjamini–Hochberg-adjusted P values, with detailed results provided in Supplementary Table S5.

**FIGURE 12:**
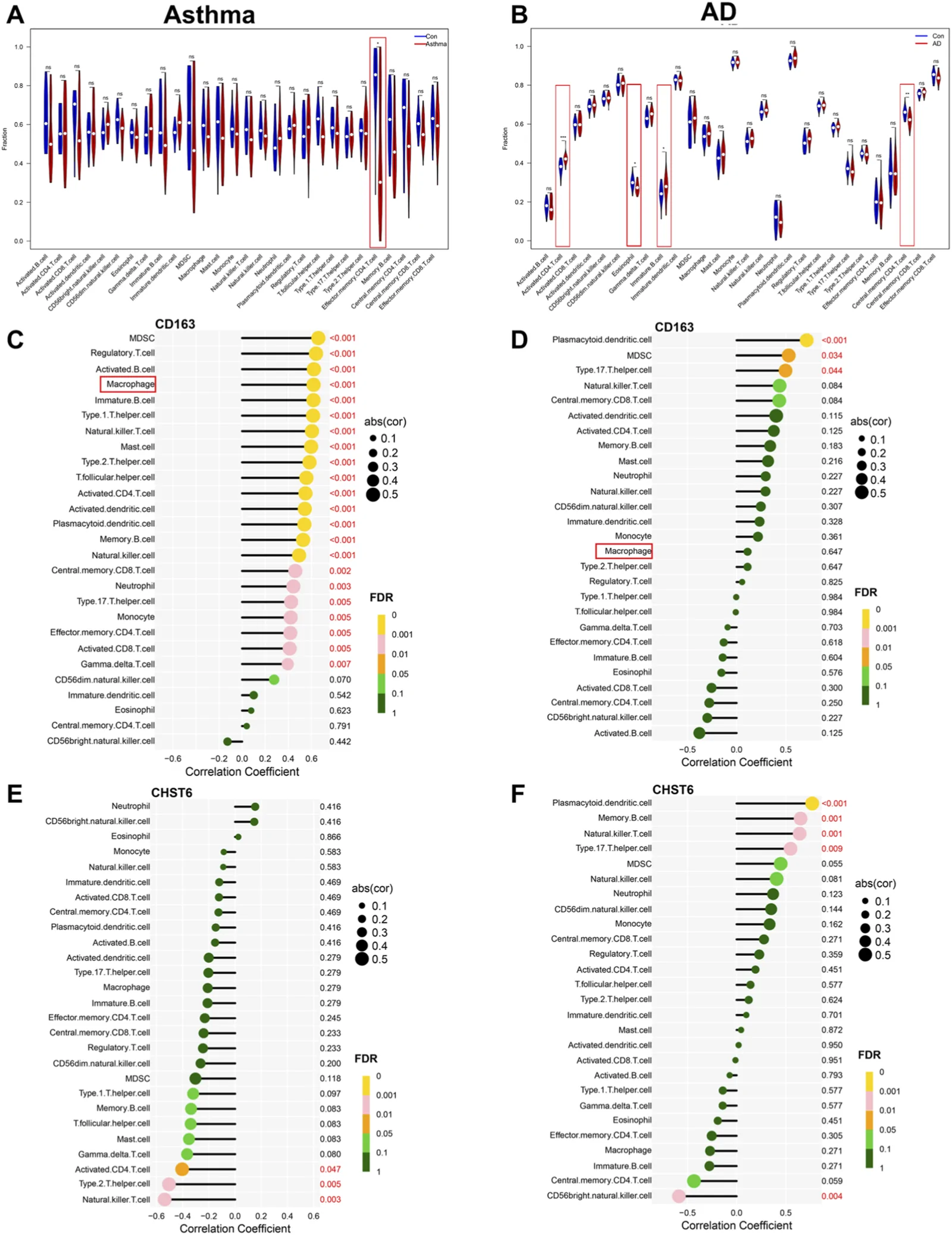
ssGSEA-based immune-cell signature analysis in asthma and AD. **(A,B)** Violin plots comparing immune-cell signature enrichment scores between disease and control samples in asthma **(A)** and AD **(B)**. **(C,D)** Correlation analysis between CD163 expression and immune-cell signatures in asthma **(C)** and AD **(D)**. **(E,F)** Correlation analysis between CHST6 expression and immune-cell signatures in asthma **(E)** and AD **(F)**. Statistical significance was determined using Benjamini–Hochberg-adjusted P values. For violin plots, *FDR <0.05, **FDR <0.01, and ***FDR <0.001.

### 3.14 Expression of genes in single cells

Single-cell analysis identified distinct cell populations in the AD brain and asthma-related datasets (Figures 13A,B). CD163 showed a predominantly myeloid-associated pattern, with highest expression in microglia in the AD dataset and monocytes in the severe-asthma PBMC dataset (Figures 13C,D; Supplementary Table S8). In A^+^T^+^ microglia, 5.75% of cells expressed CD163, with a mean expression of 0.116, compared with 1.90% and 0.044 in A^−^T^−^ controls. The cell-level difference was significant *(P* = 0.0047; Supplementary Table S9), although CD163 was also prominent in A^+^T^−^ microglia. At the donor level, CD163 remained directionally higher in A^+^T^+^ microglia (1.47-fold) but was not significant (*P* = 0.53; Figure 13E; Supplementary Table S10). A similar positive but nonsignificant donor-level pattern was observed in severe-asthma monocytes versus healthy controls (*P* = 0.56). CHST6 showed a different pattern. In the AD dataset, expression was generally low but relatively higher in astrocytes and oligodendrocytes (Figures 13C,D). CHST6 was lower in A^+^T^+^ than A^−^T^−^ oligodendrocytes at the cell level (*P* = 4.1 × 10^−4^), whereas the donor-level difference was not significant (*P* = 0.18; Figure 13E; Supplementary Tables S9–S10). In the airway epithelial dataset, CHST6 was predominantly detected in ciliated and club cells and increased after IL-13 stimulation in club cells (*P* = 2.4 × 10^−6^) and ciliated cells (*P* = 0.030; Supplementary Table S11). Given the two-donor design, these epithelial findings were considered exploratory. Overall, the single-cell data support myeloid-associated localization of CD163 and context-dependent localization of CHST6 rather than a shared cell-type-specific mechanism between asthma and AD.

**FIGURE 13:**
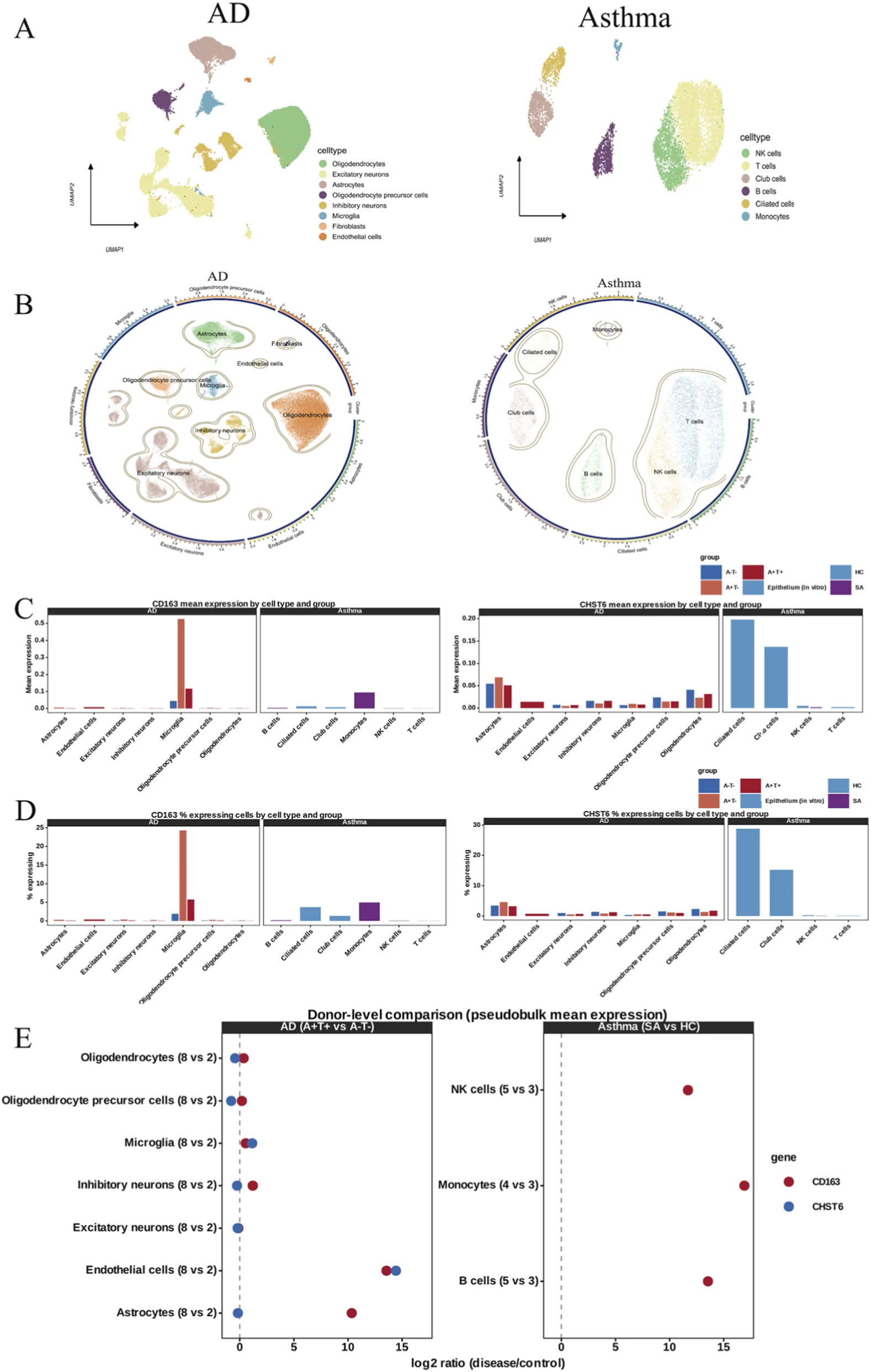
Cell-type-specific expression of CD163 and CHST6 in AD- and asthma-related single-cell datasets. **(A)** UMAP visualization of annotated cell populations in AD and asthma-related datasets. **(B)** Circular visualization of the corresponding cell clusters. **(C)** Mean expression of CD163 and CHST6 across cell types and groups. **(D)** Percentage of cells expressing CD163 and CHST6 across cell types and groups. **(E)** Donor-level pseudobulk comparison of CD163 and CHST6 expression between A^+^T^+^ AD and A^−^T^−^ controls and between severe asthma (SA) and healthy controls (HC). Donor numbers are indicated in the figure.

## 4 Discussion

Growing evidence links asthma to an increased risk of AD (Bartels et al., 2024; Chen et al., 2014; Peng et al., 2015; Rosenkranz et al., 2022), supporting the hypothesis that systemic inflammation may represent a shared factor in both conditions. To explore this, we integrated DEGs and WGCNA analyses of lung and brain tissues, identifying 24 common genes. Functional enrichment indicated involvement in immune pathways as well as metabolic processes, including vitamin metabolism, the TCA cycle, and MAPK signaling (de Matos Rodrigues et al., 2024; Hescot et al., 2022; Puig-Kröger et al., 2009; Pomin, 2015), suggesting coordinated immune dysregulation and metabolic disturbance.

CD163, a macrophage scavenger receptor shed during inflammation as soluble sCD163 (Hintz et al., 2002), was identified as a candidate gene of interest. CD163 is a monocyte/macrophage-associated hemoglobin scavenger receptor with anti-inflammatory and immunoregulatory properties (Plevriti et al., 2024). Previous studies have implicated CD163 in both diseases: in asthma, it contributes to M2 polarization, Th2 immunity, and airway remodeling (Zhi et al., 2017); in AD, increased CD163 expression has been reported in AD brain regions, with CD163-positive cells observed around Aβ plaques and damaged vessels (Yang et al., 2022; Chen et al., 2024). However, previous studies reported reduced cell-surface CD163 expression on alveolar macrophages (Dai et al., 2016) and lower CD163 mRNA expression in sputum-derived macrophages from patients with asthma (Staples et al., 2012). This apparent discrepancy may reflect differences in cell type, tissue compartment, measurement method, disease stage, or experimental model. The cited studies assessed macrophage-specific CD163, whereas our transcriptomic and mouse experiments were based mainly on bulk tissues, where expression may also reflect changes in immune-cell composition. Moreover, the MR analyses represent genetically predicted CD163 expression or circulating protein levels rather than direct measurements of disease-state expression. Therefore, the positive MR estimates and increased CD163-related signals observed in our study should not be interpreted as evidence that CD163 is uniformly upregulated across macrophage populations or acts as a pathogenic risk factor. Instead, they suggest context-dependent CD163-associated immune alterations that require further functional investigation. The established macrophage biology of CD163 itself is not a novel finding of this study; rather, our contribution lies in integrating CD163-associated signals across asthma- and AD-related datasets.

In contrast, CHST6 represented a relatively understudied candidate in this context of asthma and AD. Its canonical function is the sulfation of corneal keratan sulfate (KS) (Focus on Molecules, 2006). However, emerging evidence suggests that CHST6 may have disease-related roles beyond the cornea. CHST6 has been associated with glioma prognosis (Zhao et al., 2025), and recent studies have also placed CHST6 within AD-related molecular contexts. Wang et al. identified CHST6 as part of a glucose-metabolism-related diagnostic signature (Wang et al., 2023a), while emerging evidence has highlighted glycosylation-related alterations as a potentially relevant feature of AD (Shi et al., 2026). These observations provide indirect support for further investigation of CHST6 in AD, but do not establish a mechanistic role for CHST6 in asthma–AD comorbidity.

Firstly, CD163 and CHST6 were identified as cross-disease candidate genes by intersecting independently derived asthma and AD transcriptomic results. Because the discovery datasets originated from different tissues and platforms, their overlap should not be interpreted as evidence of a shared tissue-specific mechanism. Instead, these genes may reflect convergent inflammatory or disease-associated transcriptional changes across distinct tissue environments. Their consistent expression patterns in independent validation datasets support their reproducibility as cross-disease candidates, although their biological significance is likely context dependent. CD163 and CHST6 also showed moderate individual diagnostic performance (AUC ≤0.80), whereas their combined model achieved improved discrimination (AUC >0.87). However, this model has not been validated for prospective prediction of AD in patients with asthma and should therefore be considered exploratory rather than clinically applicable.

Secondly, we examined peripheral-blood datasets to distinguish potential systemic signals from tissue-local associations. CD163 showed concordant expression changes in peripheral-blood datasets from asthma and AD. Increased circulating sCD163 has been associated with asthma severity (Periyalil et al., 2015), while CD163 is expressed in microglia and infiltrating macrophages in AD (Pey et al., 2014). These findings support CD163 as a potential systemic immune-associated candidate but do not establish peripheral-to-central immune communication or a direct disease-promoting effect. Nevertheless, these observations remain transcriptomic associations between the two diseases. In contrast to CD163, CHST6 was not consistently detected in the available AD peripheral-blood dataset. Therefore, the current evidence does not support CHST6 as a circulating or systemic marker, and we interpret it more cautiously as a tissue-associated candidate. In particular, the inconsistent detection of CHST6 in peripheral blood limits its current feasibility as a blood-based biomarker, and its clinical relevance requires validation in accessible biospecimens and prospective cohorts.

Thirdly, SHAP analysis identified CD163 as a major contributor to AD prediction, with higher expression associated with increased disease probability. Given that asthma is characterized by elevated systemic CD163 (Periyalil et al., 2015), this finding supports CD163 as a cross-disease immune-associated candidate; however, it does not establish that peripheral CD163 alterations contribute directly to neurodegeneration.

Fourthly, ssGSEA revealed tissue-dependent associations between CD163/CHST6 expression and immune-cell signature enrichment. The associated immune signatures differed between the asthma and AD datasets, which may reflect differences in tissue composition and inflammatory context involving peripheral–central immune crosstalk (Wang et al., 2023b) and monocyte-derived brain infiltration (Muñoz-Castro et al., 2023). However, ssGSEA provides indirect transcriptomic estimates rather than direct measurements of immune-cell abundance, and these correlations do not establish that CD163 or CHST6 regulates immune-cell infiltration or mediates peripheral–central immune communication. Further validation using cell-specific or orthogonal experimental approaches is required.

Finally, single-cell RNA sequencing provided cell-type-specific context for the candidate genes. CD163 was predominantly localized to microglia in the AD brain and monocytes in severe-asthma PBMCs, consistent with its established monocyte/macrophage biology (Plevriti et al., 2024), the recognized role of microglia in CNS immune processes (Norris and Kipnis, 2019), previous reports of CD163-associated myeloid alterations in AD (Pey et al., 2014), and altered circulating sCD163 in asthma (Periyalil et al., 2015). However, the corresponding donor-level disease–control differences were not statistically significant; therefore, these findings primarily support cellular localization rather than disease-specific upregulation or direct peripheral-to-central immune communication. CHST6 showed a distinct, context-dependent distribution. In the AD brain, its expression was relatively low across cell populations but detectable in glial populations, including astrocytes and oligodendrocytes. Although oligodendrocytes play important roles in myelin homeostasis and AD pathophysiology (Kedia and Simons, 2025), our revised analysis did not support preferential or disease-specific CHST6 enrichment in oligodendrocytes. In contrast, CHST6 was more prominent in ciliated and club cells in the airway epithelial model and increased following IL-13 stimulation. Given the central role of IL-13 in type 2 airway inflammation (Howell et al., 2023) and previous transcriptomic studies identifying CHST6 as an AD-associated candidate (Wang et al., 2023a; Shi et al., 2026), these findings suggest tissue- and context-dependent regulation of CHST6. Overall, the single-cell data provide cellular-context information but do not establish a shared cellular or mechanistic pathway linking asthma and AD.

This study has several limitations. Firstly, the lack of detailed clinical information in public datasets may introduce confounding factors. Secondly, the relatively small sample sizes may not fully capture disease heterogeneity. The lower performance of the LR model in external validation (≈0.77 vs. >0.87 in the training cohort) further indicates limited generalizability and the need for validation in larger independent cohorts. Thirdly, differences in cohort composition, tissue source, and platform may affect reproducibility. Because asthma discovery was based on airway tissue whereas AD discovery was based on brain tissue, overlapping genes may partly reflect general inflammatory or cell-composition signals rather than disease-specific shared mechanisms. WGCNA was performed separately within each normalized dataset without cross-platform pooling, although residual within-cohort technical variation cannot be excluded. In addition, the single-cell disease–control analyses were limited by small numbers of biological donors, particularly the 2 A^−^T^−^ normal donors in GSE243292 and the two donors in the GSE145013 epithelial model; accordingly, cell-level comparisons were interpreted cautiously and donor-level analyses were considered exploratory. Finally, the animal experiments used separate asthma and AD models with a small sample size (*n* = 3 per group) and provide only exploratory expression-level validation. They do not demonstrate asthma-induced AD pathology or CD163/CHST6-mediated lung–brain immune crosstalk. Further validation in larger cohorts and mechanistically relevant experimental models is required.

## 5 Conclusion

In conclusion, our study identifies CD163 and CHST6 as candidate molecular links between asthma and AD. CD163 may reflect a systemic immune component associated with neuroinflammation, while CHST6 may reflect tissue- and context-dependent cellular changes. These findings provide a preliminary framework for understanding asthma–AD comorbidity and highlight candidate biomarkers that warrant further investigation in prospective studies of asthma patients with longitudinal cognitive outcomes.

## Data Availability

The original contributions presented in the study are included in the article/Supplementary Material, further inquiries can be directed to the corresponding author.
