# Supplementary material for: Immune-related molecular links between asthma and Alzheimer’s disease identified by multi-omics, machine learning and single-cell analysis
Source: Front Cell Dev Biol. 2026 Sep 28;14:1907664. doi: 10.3389/fcell.2026.1907664 (PMC13655240; doi:10.3389/fcell.2026.1907664)
Supplement: Supplementary file 1 [file DataSheet1.docx]

**Supplymental figure S1** ROC curve demonstrating the machine learning models’ diagnostic performances in Asthma and AD.

**Supplymental figure S2. Sensitivity analyses of the associations between genetically predicted CD163 expression or plasma protein levels and AD- and asthma-related phenotypes.** (A–C) CD163 gene expression and Alzheimer disease: MR scatter plot (A), funnel plot (B), and leave-one-out analysis (C). (D–F) Plasma CD163 levels and dementia in Alzheimer disease: MR scatter plot (D), funnel plot (E), and leave-one-out analysis (F). (G–I) CD163 gene expression and the asthma-and-allergy phenotype: MR scatter plot (G), funnel plot (H), and leave-one-out analysis (I). (J–L) Plasma CD163 levels and allergic asthma: MR scatter plot (J), funnel plot (K), and leave-one-out analysis (L). Scatter plots show estimates obtained using different MR methods, funnel plots provide a visual assessment of asymmetry among individual SNP estimates, and leave-one-out analyses assess the influence of individual genetic instruments on the overall estimate.

**Supplymental figure S3. Single gene GSEA enrichment analysis of key shared diagnostic genes.** GO and KEGG enrichment analyses for CD163 and CHST6 using GSEA, displaying the top ten enriched terms.
